# Supplementary material for: Content-rich biological network constructed by mining PubMed abstracts
Source: BMC Bioinformatics. 2004 Oct 8;5:147. doi: 10.1186/1471-2105-5-147 (PMC528731; doi:10.1186/1471-2105-5-147)
Supplement: Additional File 2 — The original results of the above study (non-essential files are deleted to keep the file size under the limit set by BMC bioinformatics). [file 1471-2105-5-147-S2.bz2 › chilibotAdditionalFile2/dip10/306ID9117348E1304/html/CD44_CD9.html]

 


 **CD44** and **CD9** 
  
Found 109 abstracts in PubMed, retrieved 10.  
 

 What does Google say? 
 PDF only 
| .edu only 

---

**Interactive relationship** (e.g. stimulation, inhibition, etc)

**Non-interactive relationship** (e.g. studied together, co-existance, homology, etc.)

- Nonetheless, Pgp  [ **CD44** ]  and MRP1  [ **CD9** ]  are detected in a significant proportion of untreated breast cancers on average 40 and 50% respectively, by immunohistochemistry, without a clear and consistent association with cancer stage.  Ref: 12653670 Endocr Relat Cancer, 2003
- Based on their immunophenotype, the cells are relatively homogeneous, with shared expression of integrin beta 1, the hyaluronate receptor  [ **CD44** ] , and the tetraspan protein  **CD9** , among other markers.  Ref: 12880371 Expert Opin Biol Ther, 2003
- The findings indicate that the in vivo expression of LRP, but not of Pgp  [ **CD44** ]  and MRP 1  [ **CD9** ] , is induced by chemotherapeutic treatment in rhabdomyosarcomas.  Ref: 12612883 Hum Pathol, 2003
- The expression, of multidrug resistance MDR associated proteins PGP  [ **CD44** ] , LRP, MRP1  [ **CD9** ]  was also analyzed.  Ref: 12801292 Eur J Haematol, 2003
- Paraffin embedded samples of 13 pairs of primary untreated rhabdomyosarcomas and their residual, recurrent, or metastatic lesions after chemotherapy were assessed for expression of MDR proteins, including P glycoprotein Pgp  [ **CD44** ] , multidrug resistance associated protein MRP 1  [ **CD9** ] , and lung resistance related protein LRP.  Ref: 12612883 Hum Pathol, 2003
- No correlation was found between Pgp  [ **CD44** ] , MRP1  [ **CD9** ] , and BCRP expression and response to chemotherapy and survival.  Ref: 12644825 Br J Cancer, 2003
- These include MDR1 P glycoprotein, PgP  [ **CD44** ] , the multidrug resistance related proteins MRP1  [ **CD9** ]  5, and lung resistance protein LRP.  Ref: 12699350 Curr Drug Targets, 2003
- Data obtained are presented in concurrence with Clusters of Thymic Epithelial Staining CTES classification for various monoclonal antibodies recognizing CD antigens CD1, CD1a, CD6,  **CD9** , CD14, CD16, CD29, CD30, CD32,  **CD44** , CD45RB, CD47, CD48, CD49a, CD49b, CD49c, CD49d, CD49e, CD49f, CD51, CD53, CD54, CD56, CD57, CD63, CD85, CD95, CD98, CD102, CD103, CD106, CD109, CD146, CD147, CD148, CD151, CD152, CD158a, CD158b, CD164, CD165, CD166 and for monoclonal antibodies 1B1 G7, A4, BD46, BLTZ, HP1C5, IND.64, M72, WU947 whose specifities are not yet defined.  Ref: 12725440 Ann Anat, 2003
- Pgp  [ **CD44** ]  or MRP 1  [ **CD9** ]  expression did not change significantly, but LRP expression increased significantly after chemotherapy.  Ref: 12612883 Hum Pathol, 2003
- To address whether multidrug resistance protein MRP affects GO susceptibility we characterized Pgp  [ **CD44** ] , MRP1  [ **CD9** ] , and MRP2 expression in CD33 cell lines and CD33 AML samples and analyzed the effect of the Pgp  [ **CD44** ]  inhibitor cyclosporine CSA and the MRP inhibitor MK 571 on GO induced cytotoxicity.  Ref: 12689934 Blood< MedlineTA>Blood, 2003
- However, in the last decade considerable attention has been dedicated to the role played by membrane transporter proteins belonging to the ATP binding cassette protein superfamily, and in particular by the MDR1 product P glycoprotein Pgp  [ **CD44** ]  and the multidrug resistance protein MRP1  [ **CD9** ] .  Ref: 12653670 Endocr Relat Cancer, 2003
- Paraffin embedded sections from 56 previously untreated patients with metastatic TGCT were immunostained for Pgp  [ **CD44** ] , MRP1  [ **CD9** ] , BCRP, and LRP.  Ref: 12644825 Br J Cancer, 2003
- PGP  [ **CD44** ] , LRP, and MRP1  [ **CD9** ]  expression at onset and at first relapse was low.  Ref: 12801292 Eur J Haematol, 2003
- This study was undertaken to investigate the expression and predictive value for outcome of multidrug resistance associated MDR proteins P glycoprotein Pgp  [ **CD44** ] , MRP1  [ **CD9** ] , BCRP, and LRP, in advanced testicular germ cell tumours TGCT.  Ref: 12644825 Br J Cancer, 2003
